# Digital studying in times of COVID-19: teacher- and student-related aspects of learning success in german higher education

**DOI:** 10.1186/s41239-023-00382-w

**Published:** 2023-03-03

**Authors:** Ole Engel, Lena M. Zimmer, Markus Lörz, Elisabeth Mayweg-Paus

**Affiliations:** 1 Department for Higher Education, Institute of Educational Studies, Humboldt-Universität Zu Berlin, Unter Den Linden 6, 10099 Berlin, Germany; 2 Office of Vice President for Academic Affairs, Humboldt-Universität Zu Berlin, Unter Den Linden 6, 10099 Berlin, Germany; 3 Department of Educational Governance, Leibniz Institute for Research and Information in Education, Rostocker Straße 6, 60323 Frankfurt Am Main, Germany

**Keywords:** Higher education, Learning success, Digital teaching, Study situation, COVID-19

## Abstract

In the wake of COVID-19, study conditions in Europe have changed dramatically. To limit contact between students and teachers, since March 2020 teaching has largely taken place digitally (remotely via digital means) and in private. Because the success of digital learning likely relies on many factors beyond good digital infrastructure conditions, this article focuses on which aspects, at both the teacher and the student levels, promote digital learning success. The large-scale student survey “Studying in Times of the Corona Pandemic” conducted at German universities and universities of applied sciences in the summer semester of 2020 offers data on how COVID-19 has affected several aspects of university studying in Germany. Here, we consider this data within the theoretical framework “theory of transactional distance” introduced by Moore (in: Moore (ed) Handbook of distance education, Routledge, 2018), according to which the success of digital teaching is influenced by dialogue, structure, and learner autonomy. Based on various regression analyses, our results show that several (digital) framework conditions must be created on both the teacher and student levels to achieve sufficient digital learning success. In this sense, our findings provide guidance on which aspects institutions of higher education should focus on when developing or updating their digitalization strategies. In accordance with collaborative learning approaches a key factor for learning success appears to be enabling peer-to-peer interactions. This finding supports our prediction that the possibility of engaging in interactive learning activities is crucial for students’ learning experience, as it might reduce the perception of transactional distance and allow for social exchange. The strongest predictor of students’ learning success turned out to be the (perceived) digital competencies of the teachers. This finding clearly emphasizes that teachers must be qualified to address the very specific challenges of teaching in digital contexts and indicates that universities may need to implement more teacher qualification programs.

**Supplementary Information:**

The online version contains supplementary material available at 10.1186/s41239-023-00382-w.

## Introduction

The COVID-19 pandemic led to massive changes in the design of teaching at European universities. Since the summer semester of 2020, teaching in European higher education institutions has been transformed from an almost exclusively analog process to an almost exclusively digital one. Up until this point, digitalization in Germany had not been strongly developed compared to other European countries (European Commission, 2020); this situation changed dramatically during the course of COVID-19. According to a nationwide survey of students in German higher education, while most courses offered in the winter semester of 2019/20 were exclusively analog, almost all courses offered in the summer semester of 2020 were mostly or exclusively digital (Lörz et al., 2020). Throughout Germany, universities have extensively implemented campus management and learning management systems, and the first findings indicate that students, as well as teachers, are largely satisfied with the university-supplied digital equipment, though they often must use their own private digital devices for teaching and learning. Regarding university administration, it seems that German universities are well prepared for digital teaching during the pandemic (Autorengruppe Bildungsberichterstattung, 2020).

Although almost all universities offer digital teaching programs and only a few students have reported that their courses have been canceled (Lörz et al., 2021), the question remains to what extent the pandemic-related short-term shift to online teaching has met the standards of high-quality online teaching. High-quality online instruction should be based on research-backed theories and principles for didactic planning and design (Adedoyin & Soykan, 2020). Many distinct factors for high-quality online instruction certainly exist at the teacher level, but those at the student level also need to be considered (Cheawjindakarn et al., 2012). The very sudden and specific changes caused by the pandemic may allow us to better understand how these factors, which are directly related to the educational setting, affect the perceived quality of digital teaching and learning. Thus, here we concentrate on teacher-centered and student-centered factors that might impact students’ satisfaction with how online teaching was realized. The teaching in the summer semester 2020 can be described as so-called emergency remote teaching (Hodges et al., 2020). In this respect, it is not comparable with well-founded, planned online teaching. Meta-analyses on digital learning have been based almost exclusively on experimental studies (Hattie, 2009; Schaumburg & Prasse, 2018), while field research has hardly been systematically incorporated into the theory development.

Concerning teacher-related aspects, Watermeyer et al. (2021) pointed out that less than half of the lecturers in the United Kingdom felt prepared to deliver online learning, teaching, and assessment. Accordingly, the transition to online teaching has been dysfunctional, and lecturers’ pedagogical roles and personal lives have been disturbed. Based on interviews with university teachers in Sweden, some key influencing factors for successful online teaching include social engagement and close student–teacher relationships. Both factors positively impact students’ motivation, satisfaction, and (indirectly) learning outcomes (Jensen et al., 2020). In Germany, the main criticisms about teaching that arose during the pandemic have mainly been in subjects that heavily emphasize excursions or practical exercises, because such events were often canceled and not replaced with digital options. In addition, students mentioned problems due to a lack of access to infrastructures, such as study rooms or libraries, as well as the general lack of opportunities for interacting with other students and teachers (Horstmann et al., 2021).

Regarding student-related aspects, Aristovnik et al. (2020) showed for several countries that students’ performance in the digital context was especially challenged by deficient digital competencies and a perceived higher workload. This comparatively higher workload was also documented for German students during the digital semester (Traus et al., 2020). Furthermore, Flores et al. (2022) reported that crucial factors for students’ successful participation in online learning include individual self-regulatory and socio-emotional competencies as well as adequate digital equipment. Händel et al. (2022) surveyed students’ readiness for digital learning in higher education during the COVID-19 pandemic, and they found via a cluster analysis that two groups emerged—one group (57%) that was highly ready for digital learning and one group (43%) that was not—based on students’ available equipment, earlier experience, self-reported competencies, and information sharing behavior.

In summary, several studies regarding teacher- and student-related aspects have already examined the conditions of digital teaching in higher education during the COVID-19 pandemic. With regard to the student-related aspects, evidence suggests that learning success depends on digital, self-regulatory, and socio-emotional competencies as well as access to digital equipment. Yet, significantly less is known about teacher-related aspects, except that the shift to digital teaching has been a major challenge and that learning success is fostered by social engagement and close student–teacher relationships. In general, as most of the above studies were developed empirically, they remain mostly descriptive; thus, we do not yet know which aspects at the teacher and the student levels contribute to learning success and to what extent. By applying multivariate analyses, the present study strives to gain deeper insight into the role of teacher-related and student-related aspects in students’ learning success. Our analysis offers the opportunity to incorporate the experiences and assessments of over 18,000 students. Further, our analysis also points to practical implications for teaching, i.e. which factors should be taken into account by teachers to conduct online teaching successfully. In the recent summer semester from April until July 2022, a large part of university teaching has taken place face-to-face again. However, in addition to the uncertainty about the further course of the pandemic, the proportion of digital teaching has increased significantly compared to the time before the pandemic. Accordingly, a large number of teachers are facing the question of an adequate and well-founded design of online teaching. The study presented provides insight into which student- and teacher-related aspects are of decisive importance for future digital teaching.

## Theoretical framework

Researchers have long investigated factors that influence learning success in the context of online education, but no overarching theory has yet been developed (Krammer et al., 2020a, 2020b). Nevertheless, different theoretical approaches to online education have been discussed, and they mostly relate to the major learning theories of cognitivism, behaviorism, and constructivism (Picciano, 2017). In addition to approaches dealing with collaborative online learning (Harasim, 2017) and the community of inquiry (Cleveland-Innes et al., 2018), another approach is the theory of transactional distance. This theory, developed by Moore in the 1970s, can be classified as the first theory of distance education (Moore, 1973), and it is still one of the most widely used theoretical approaches to online education (Moore, 2018); thus, it forms the theoretical framework for our analysis. However, the aim of this work is not to develop a theory-testing model but to use the theory of transactional distance to systematize and identify important influencing factors for successful online teaching.

First used by John Dewey, the term *transaction* refers to the “interplay among the environment, the individuals and the patterns of behavior in a situation” (Moore, 2018, p.33). Accordingly, transactional distance describes the gap in what a student understands about reality and the understanding of that same reality by the teacher. Based on this concept, Moore defines *distance education* as “the methodology of structuring courses and managing dialogue between teacher and learner to bridge that gap through communication technologies” (Moore, 2018, p.34). For successful distance education, the transactional distance can be minimized and the gap between learner and teacher can be bridged through (1) dialogue, (2) structure, and (3) learner autonomy (Moore, 2018).

*Dialogue* is strongly influenced by communication technology. In this sense, dialogues between teachers and learners can be created by asynchronous teaching with recorded materials and possibilities for interaction by, for example, email. Yet, interactive communication technologies hold much potential; web conferences, for example, enable intensive, personal, individual, and dynamic dialogue. Other important factors are the frequency with which opportunities for communication arise as well as the physical environment in which students learn and teachers teach (Moore, 1997). Overall, the more dialogue that can take place, the lower the transactional distance, and, finally, the higher the potential for successful distance education.

*Structure* refers to the rigidity or flexibility of the educational aims, teaching strategies, and evaluation methods. A very high degree of structure is understood as a strictly specified lesson design with little possibility of deviation. A higly structured learning environment is based on explicit learning objectives and every step of the lesson is organized based on an accurate scripted plan. Highly standardized digital learning does not give students the possibility to determinate their own learning goals und develop their own learning pathways and strategies.

In contrast, a less structured learning setting is also based on a well planned lesson but it is at the same time responsive to a learner’s individual needs and preferences. The teacher acts more as a learning guide than as a learning instructor (Moore, 2018). With regard to the use of communication technologies, this means that web conferencing, for example, offers the possibility of less structured learning environments in the form of teacher-learner dialogues and transactional interplay. In contrast, recorded material for reception does not offer this possibility and thus corresponds more closely to the model of highly standardized learning environments (Moore, 1997). In summary, the lower the degree of structure, the lower the transactional distance, and, finally, the higher the potential for successful distance education.

*Learner autonomy*, in contrast to structure and dialogue, is not about how the teacher designs distance education but instead focuses on the student. Learner autonomy is about a student’s ability to shape their learning process in a self-determined way (Moore, 2018). In the 1970s, this concept challenged the hegemony of the behaviorist approach, which was primarily based on the systematic design with maximum teacher control of the learning process. The concept of learner autonomy has illustrated that when applying behaviorist forms of teaching, university teachers were not using the potential of independent learning. Learning in school can still be described as highly dependent, so in higher education, the ability to learn independently must first be developed. Accordingly, learner autonomy describes the extent to which the learner, within the teaching–learning relationship, takes responsibility for the learning goals, learning aims, and evaluation decisions (Moore, 1997). In summary, the higher the learning autonomy, the lower the transactional distance, and, finally, the higher the potential for successful distance education.

Based on these theoretical assumptions, we can distinguish between the teacher-related and student-related factors at play in digital learning. First, because structure and dialogue are mostly initiated by teachers, they can be classified as teacher-related factors; by contrast, learner autonomy depends primarily on the experience and competencies of the students, so it can be categorized as a student-related factor. Looking at both types of factors, here we examine how learning success is influenced by the general setting in which digital teaching takes place, the interaction opportunities, and teachers’/students’ digital competencies.

### Teacher-related aspects of learning success

Structure and dialogue are strongly related to communication technologies. For example, videoconferencing and webinars open up the possibility for a flexible structure and offer a variety of opportunities for dialogue. Further, synchronous teaching enables real-time interaction, instant feedback, and new possibilities for collaboration, and teachers and students can express themselves through audio, visual, and verbal communication with others. In this sense, synchronous digital teaching formats come much closer to face-to-face teaching than asynchronous teaching formats. As learning success is influenced by the interactions between learners and their shared experience (Correia et al., 2020), a key factor that has been mentioned in subjectively experienced learning success is videoconferencing, because it enables meeting, exchange, input, and screen sharing (Krammer et al., 2020a, 2020b). Additionally, videoconferencing can promote educational collaboration and the emergence of learning communities (Carrillo & Flores, 2020; Martin, 2005). Thus, social presence plays a key role in successful online teaching and learning. Along these lines, students’ abilities to perceive others in an online environment have a huge influence on their motivation and participation as well as on actual and perceived learning success (Richardson et al., 2017). Furthermore, video conferencing facilitates learner-centered engagement, in that when learners create different conference rooms (e.g., break-out rooms), they can switch between different forms of interacting with other students and with the lecturer (Smyth, 2005). Hence, we developed the following hypothesis:

**H 1.1:** The more frequently synchronous digital teaching takes place, the higher students’ learning success.

The second teacher-related factor is whether the teacher provides possibilities for active interaction. Besides promoting dialogue, facilitating interaction possibilities indicates a flexible teaching strategy.

Tomasello (2011) pointed out that humans’ social constitution is special because of our ability to cooperate, communicate, and transmit social and cultural information to each other; that is, we learn through each other. Transferring this concept to distance education, the most important ways to foster active interaction include offering opportunities for learners to participate in group discussions, change their perspectives, introduce their ideas, and interact and respond to different ideas. Allowing students to draw on ideas from different points of view not only supports creativity but also enables deep learning (Harasim, 2017; Rennar-Potacco & Orellana, 2018). In contrast, it appears that a lack of student interaction and support tends to result in students dropping out.

It can thus be assumed that a relevant factor for students’ learning success in digital teaching is whether teachers offer the opportunity for active interaction. Against the background of these considerations, the following hypothesis was developed:

**H 1.2:** The higher the proportion of online courses with possibilities for active interaction, the higher students’ learning success.

The third factor focuses on teachers’ digital competencies. Basilotta-Gómez-Pablos et al., (2022) show in their review that there is a growing interest in knowing the state of digital literacy among higher education teachers and the set of knowledge, skills, and attitudes that a teacher needs to use technology effectively. Regarding the definition of teachers`digital competencies the review points out a consensus that teachers must have didactic and technological skills that enable them to use digital technologies in higher education. In addition to technological, informational, multimedia, communicative, collaborative and ethical knowledge, pedagogical-didactic skills are particularly important for the integration of information and communication technologies in educational practice (Basilotta-Gómez-Pablos et al., 2022). The European Framework for Digital Competence of Educators points out different levels and forms of digital compentences in education In our context digital competencies refer to the area of teaching and learning in higher education, which includes four levels. Beside the teaching process itself, it includes the guidance to use digital technologies to enhance the interaction with learners, the competence to foster and enhance learner collaboration and the knowledge to promote self-regulated learning with digital devices (Punie & Redecker, 2017). To create a flexible course structure as well as to enable dialogue, teachers must have these necessary digital competencies and knowledge. Learning can be understood as a social process that takes place through contact and discourse with competent teachers. In the context of digital learning, this not only refers to teachers’ professional competencies but also their digital competencies. To make the most use of synchronous videoconferences and their opportunities for interactive exchange, teachers need a sufficient understanding of interactive tools and technologies. As such, teachers should understand the current and future functionality and capabilities of learning technologies as well as their impact and implications (Harasim, 2017). Against this background, we assume:

**H 1.3:** The higher students’ satisfaction with teachers’ digital competencies, the higher students’ learning success.

### Student-related aspects of learning success

A basic requirement for learners’ autonomy and self-determined learning is that students' living situations must be suitable for digital teaching. During the COVID-19 pandemic, students’ housing conditions played an even greater role, as alternative places for participating in digital teaching were unavailable. Housing conditions influence the possibility of dialogue since dialogues depend on an appropriate physical environment (Moore, 1997). Physical space is an important part of learning environment and might support or hinder the learning process, The ability to structure social behavior in time and space, the indoor environment or the acoustics are only examples of important aspects of the housing learning environment. Sometimes the physical infrastructure is even described as a kind of third teacher (Arnou et al., 2020). International-comparative studies about digital learning in higher education during COVID-19 point out the home learning environment as one of the most significant factor (Cranfield et al, 2021).

Thus, for students to successfully participate in digital learning, they must have a good, stable Internet connection, opportunities to withdraw, and a quiet learning environment. Accordingly, it can be assumed that the described potential of synchronous teaching can only be exploited if these prerequisites are met, which leads to the following hypothesis:

**H 2.1** The worse suited a student’s housing situation is for digital learning, the lower the student’s learning success.

A second important indicator for learner autonomy is exchanging between learning communities. Learning how to shape one’s own learning process in a self-determined way is challenging, and to meet this challenge, students benefit from exchanges and peer collaborations that take place in study groups, as such exchanges help them learn collaboratively in a self-determined way and reflect on and discuss the challenges of self-determined learning.

Students in learning communities often form self-supporting groups that exist even beyond classroom activities and help students engage actively in deeper learning processes. Thereby, collaborative engagement with others can positively affect the quality of learning, as it promotes deeper elaboration of the learning content (Mayweg-Paus et al., 2016) and students report higher intellectual gains (Molinillo et al., 2018). In this sense, interaction seems to be an important factor in students’ satisfaction, the cohesion of learning communities, and the co-construction of knowledge (Chen et al., 2018). Expanding this idea to digital learning, one can then characterize connectedness and the creation of supportive learning environments and learning communities as crucial features for successful learning in digital higher education (Carrillo & Flores, 2020). Thus, for successful learning in digital settings, online classes should offer interaction possibilities as well as opportunities for study-related exchange between students (Scull et al., 2020). Therefore, the following hypothesis was formed:

**H 2.2** The better and more developed the exchange in learning groups, the higher students’ learning success.

The third student-related factor is self-attributed digital competencies. Analogous to hypothesis 1.3, students' digital competencies form the basis for learner autonomy within distance education, and digital competencies can be understood as prerequisites for active and self-determined participation in digital teaching. Because distance learning environments require that developing and working on assignments and projects take place almost exclusively on digital devices, digital competencies are fundamental for individual and self-determined working and learning processes. The use of digital technologies in critical, collaborative and creative way forms the core of digital competence (Tzafilkou et al., 2022, European Commision 2019a). The Council on Key Competences for Life-long Learning points out the confident, critical and responsible use of and engagement with digital devices for learning. (European Commision 2019b). Park and Weng (2020) show that students perceived ICT competence has positive impacts on academic performance. Despite expertise and experience in social media, many students struggle to use digital technologies for educational purposes (Scull et al., 2020). The digital readiness of students, which is based on available equipment, e-learning experience, digital tool application, and information sharing behavior, influences students’ feelings of tension, overload, joy, worry, and loneliness, which, in turn, might also influence learning success (Händel et al., 2022). Thus, we created the following hypothesis:

**H. 2.3:** The higher a student’s self-attributed digital competencies, the higher the student’s learning success.

**Fig. 1:**
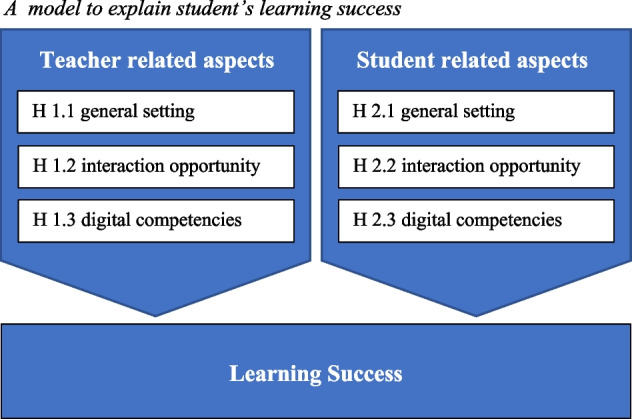
A model to explain student’s learning success

In Fig. 1 we summarize our theoretical considerations. We argue that the digital conditions of teachers and students alike influence learning success. We differentiate the degree of digitization into three components in each case, although the concrete design for teachers and students might differ. While for students the general setting is the housing situation, for teachers the general setting is the digital teaching condition. Interaction opportunity for teachers refers to the creation of possibilities for active interaction, for students it refers to the exchange that takes place in learning groups. Basically, we assume that when all three digital conditions are present for teachers and students, learning success will be highest.

## Data, variables, and methods

### Data

The hypotheses developed here were examined using a tailor-made, Germany-wide student survey (“Studying in Germany in Corona Times”) carried out in the summer semester of 2020 by the German Centre for Higher Education Research and Science Studies (DZHW) and the Research Group on Higher Education at the University of Konstanz. To gain a realistic picture of students’ situations in times of the COVID-19 pandemic, around 192,000 students were invited via email to join the online survey between June and August 2020. The data sampling was carried out in two stages. A systematic selection of 23 universities according to their distribution across federal states and by size, subject structures, and type of university was followed by a random selection of students within these institutions. Students were invited to the online survey via the contact persons at the universities. The participation in the survey was voluntary and respondents were informed about the further use of data (informed consent).^1^ The survey placed special emphasis on the hardships brought about by the crisis and included questions considering the changes between the first digital semester and the situation before the COVID-19 pandemic. The main part of the questionnaire was subjected to various longstanding pre-tests (eight expert interviews, two field-pre-tests) and resulted in large parts from previous surveys (20st and 21st social survey, Middendorff et al., 2017). However a small part of the questions with COVID-19 reference had to be developed in a short time due to the novel situation. With 28,623 completed surveys, the total response rate was about 15%. After excluding responses with missing information, our remaining analytical sample included 18,262 valid completed surveys. The main reason for this reduced analytical sample is the survey dropout towards the end of the questionnaire—with around 35 min and 91 pages it was a longer-than-average survey. To compensate for sample-related biases, the weighting is carried out along the official statistics on the composition of the student body (gender, university semester, field of study, and type of higher education institution) and normalized to the sample.

### Variables

To describe students’ learning success during the COVID-19 pandemic, we mainly drew on the students’ *perceived* learning success (dependent variable). Because the data collection had taken place in the summer semester of 2020, our analysis can provide insights into digital learning in the early phase of the COVID-19 pandemic and its impact on students’ situations. Consequently, however, we could not use long-term factors for describing learning success (e.g., competencies or grades). Thus, to gain an immediate impression of the impact of the different study circumstances on students’ learning success in the first digital semester, we drew on *students’ (subjective) satisfaction with their knowledge and competencies acquired to date*. The respondents were able to rate their satisfaction on a 5-point scale ranging from 1 “not satisfied at all” to 5 “very satisfied”.

The independent variables used to explain learning success are illustrated in Table 1. Regarding the worked-out predictors for students’ learning success, we expected effects on the teacher level (1) and effects on the student level (2): At both levels, the expected effects can further be distinguished by (1) general setting in which digital teaching takes place, (2) interaction opportunities, and (3) digital competencies.

**Table 1:** Means, standard deviations, minimum, maximum, and correlations, N = 18,262

	Variable	M (SD)	Min	Max	Corr (Spearman)
Dependent variable	Satisfaction with your knowledge and competencies acquired to date (1 *not satisfied at all* to 5 *very satisfied*)	3.1 (1.1)	1	5	–
Teacher-related predictors	**H 1.1:** Course takes place as a **videoconference/webinar **(1 *none* to 5 *all*)	3.5 (1.2)	1	5	0.11***
	**H 1.2:** Course offers active **interaction opportunities **(1 *none* to 5 *all*)	3.7 (1.1)	1	5	0.19***
	**H 1.3:** Satisfaction with **teacher’s digital competencies **(1 *not satisfied at all* to 5 *very satisfied*)	3.3 (1.0)	1	5	0.35***
Student-related predictors	**H 2.1: Living situation** is not suitable for many forms of digital learning (1 *not at all* to 5 *absolutely correct,* recoded)	3.9 (1.3)	1	5	0.22***
	**H 2.2: Exchange within study groups** during the Corona pandemic (1 *became more difficult* to 5 *became easier*)	1.8 (.96)	1	5	0.22***
	**H 2.3:** To what extent do you possess **digital competencies **(1 *not at all* to 5 *to a large extent*)	3.9 (.93)	1	5	0.12***

*p < 0.05, **p < 0.01, ***p < 0.001. *Data*: “Studying in Germany in Times of the Corona Pandemic”

To describe the general setting (digital teaching situation) on the teacher level, we drew on (H 1.1) students’ estimated *share of courses that took place as a videoconference/webinar* in the summer semester 2020 (5-point scale from 1 “none” over 3 “about half” to 5 “all”). Concerning the opportunity for direct interaction with teachers and students (H 1.2), the respondents were asked how many of their *courses offered active interaction opportunities* in the summer semester 2020 (5-point scale from 1 “none” over 3 “about half” to 5 “all”). Finally, the digital competencies on the teacher-related level were measured by (H 1.3) students’ *satisfaction with their teacher’s digital competencies* (5-point scale from 1 “not satisfied at all” to 5 “very satisfied”). As expected, all three variables on the teacher level were significantly correlated with the students’ satisfaction with their acquired knowledge and competencies (Table 1).

Regarding the general setting of digital teaching on the student level (H 2.1), we asked students to rate the statement *my living situation is not suitable for many forms of digital learning* as being on a 5-point scale of 1 “not at all correct” to 5 “absolutely correct.” We re-coded the living situation rating so that a higher number implied better suitability for digital teaching. To gain an impression of the circumstances of direct interaction on the student level (H 2.2), we focused on students’ organization in study groups. Thus, we asked whether *the exchange among study groups became more difficult or easier* during the COVID-19 pandemic (5-point scale from 1 “became more difficult” over 3 “remained the same” to 5 “became easier”). Lastly, digital competencies on the student level (H 2.3) were measured by students’ subjectively perceived own digital competencies. Hence, the students were asked *to what extent they possess digital competencies* (5-point scale from 1 “not at all” to 5 “to a large extent”). All three variables on the student level turned out to be significantly correlated with students’ satisfaction with their acquired knowledge and competencies (Table 1).

### Methods

The descriptive results indicate that the students’ perceived learning success depended on all explanatory variables. However, the extent to which these aspects influenced students’ learning success during the COVID-19 pandemic can only be determined by simultaneously considering all explanatory variables. Therefore, in the following, on the basis of the statistical program STATA we estimated *OLS regressions* with standardized predictors and—due to nested data—cluster-robust standard errors. From the regression coefficients we can see how the learning success behaves when the independent variable is increased by one unit. Thus, to test our hypotheses concerning the impact of each teacher- and student-related factor on students’ learning success, we introduced the explanatory variables stepwise into two separate regression models, one at the teacher level and one at the student level. Finally, an overall model give evidence about which factor had the most influence on students’ learning success during COVID-19. This can be seen from the size of the coefficients. In this way we can see which hypothesis is confirmed and which conditions contribute most to learning success.

In the second step of our analysis, we visualize our results of the final model and additionally estimate predicted probabilities for students’ learning success under different digital conditions on the teacher and student levels. Analyzing these scenarios provided further information about how students’ learning success was influenced by a varying degree of digitalization on both levels. Finally, the scenario analyses also allowed for conclusions to be drawn about which level (student or teacher) had a stronger impact on students’ learning success.

To reduce the risk of obtaining biased results, in all model steps we controlled for students’ gender, the field of study, university semester, and the pursued degree. As a robustness check, we additionally calculated all models as binary logistic regressions with dummy-coded predictors (see Additional file 1: Appendix).

## Results

### Determinants of learning success

In the following, we first discuss the results of stepwise OLS regressions on students’ satisfaction with their acquired knowledge and competencies. In line with our theoretical framework, the findings on the teacher level and the student level are presented separately. Thus, the results concerning the teacher-related aspects are found in Table 2, whereas the results for the student-related aspects can be found in Table 3. Finally, the overall model is presented in Table 4.

**Table 2:** Effect of the teacher-related aspects on students’ satisfaction with acquired knowledge and competencies

	M 1	M 2	M 3	M 4
Digital teaching situation	0.10*** (0.01)			0.02 (0.01)
Interaction opportunities		0.20*** (0.01)		0.10*** (0.01)
Teachers’ digital competencies			0.39*** (0.01)	0.36*** (0.01)
Constant	3.13*** (0.05)	3.10*** (0.05)	3.14*** (0.04)	3.14*** (0.04)
Observations	18,262	18,262	18,262	18,262
*R*^2^	0.018	0.041	0.131	0.139

*p < 0.05, **p < 0.01, ***p < 0.001, OLS regressions with cluster-robust standard errors in parentheses, all independent variables are standardized; models controlled for gender, subject group, university semester, and pursued degree. *Data*: “Studying in Germany in Times of the Corona Pandemic”

**Table 3:** Effect of the student-related aspects on students’ satisfaction with acquired knowledge and competencies

	M 5	M 6	M 7	M 8
Living situation	0.25*** (0.01)			0.21*** (0.01)
Exchange within study groups		0.25*** (0.01)		0.20*** (0.01)
Digital competencies			0.13*** (0.01)	0.10*** (0.01)
Constant	3.09*** (0.05)	3.08*** (0.05)	3.05*** (0.05)	3.02*** (0.06)
Observations	18,262	18,262	18,262	18,262
*R*^2^	0.060	0.057	0.022	0.101

*p < 0.05, **p < 0.01, ***p < 0.001, OLS regressions with cluster-robust standard errors in parentheses, all independent variables are standardized; models controlled for gender, subject group, university semester, and pursued degree. *Data*: “Studying in Germany in Times of the Corona Pandemic”

**Table 4:** Effect of teacher-related and student-related aspects on students’ satisfaction with acquired knowledge and competencies

		M 9
Teacher level	Digital teaching situation	0.03* (0.01)
	Interaction opportunities	0.07*** (0.01)
	Teachers’ digital competencies	0.30*** (0.01)
Student level	Living situation	0.15*** (0.01)
	Exchange within study groups	0.15*** (0.01)
	Digital competencies	0.08*** (0.01)
Constant		3.07*** (0.05)
Observations		18,262
*R*^2^		0.186

*p < 0.05, **p < 0.01, ***p < 0.001, OLS regression with cluster-robust standard errors in parentheses, all independent variables were standardized; model controlled for gender, subject group, university semester, and pursued degree. *Data*: “Studying in Germany in Times of the Corona Pandemic”

#### Teacher level

To test the impact of teacher-related aspects on students’ learning success during the digital semester, we first separately introduced the variables *share of courses that took place as a videoconference/webinar* (M 1), *courses offering active interaction opportunities* (M 2), and *students’ satisfaction with their teacher’s digital competencies* (M 3). After doing so, we checked the explanatory power of the conditions in total by considering the teacher-related aspects simultaneously (M 4). All findings are presented in Table 2.

As we can see in model 1 to model 3 (Table 2), all worked-out predictors on the teacher level had a positive, highly significant effect on students’ learning success. Model 1 indicates that the higher the share of offered videoconferences or webinars (digital teaching situation), the more satisfied students were with their learning success. Accordingly, the more courses that were offered with active interaction opportunities, the more satisfied students were with their learning success (M 2). And, finally, model 3 gives the first evidence that the more satisfied students were with their teacher’s digital competencies, the more satisfied they were with their learning success. In comparison, on the teacher level, teachers’ digital competencies seemed to be the most important predictor of students’ learning success: The regression coefficient of 0.39 indicates a relatively strong association, and, with 13.1% of the explained variance, this factor had the highest explanatory power (strong support for H 1.3). Model 4 confirms the importance of teachers’ digital competencies. Even when controlling for the share of videoconferences/webinars and the share of courses with active interaction opportunities, the regression coefficient of teachers’ digital competencies remained relatively high. Furthermore, the whole model (M 4) indicates that the mere share of courses offered as videoconferences or webinars did not have any effect on students’ learning success. Our results therefore did not support H 1.1. Instead, the most critical factor, even in times of digital teaching, seems to be that students have the opportunity to interact directly with each other and their teachers (support for H 1.2). Because the share of videoconferences/webinars lost its explanatory power when we controlled for active interaction opportunities, this might be a hint that these teaching formats often offer interaction opportunities. All in all, the teacher-related aspects explained 14% of the given variance in students’ learning success.

#### Student level

Analogous to the previous analyses, we tested the impact of students’ framework conditions on their learning success during the digital semester by first separately introducing the discussed aspects. Specifically, we tested how *students’ living situations* (M 5), the *exchange among study groups* (M 6), and *students’ digital competencies* (M 7) affected their satisfaction with their acquired knowledge and competencies. In a second step, we checked the explanatory power of the framework conditions on the student level in total by considering the student-related aspects simultaneously (M 8). The findings are presented in Table 3.

Model 5 to model 7 indicate that all worked-out predictors on the student level were significantly positively related to students’ learning success. In line with this, model 5 indicates that the more the students’ housing situations were suited for digital teaching, the higher students’ learning success (support for H 2.1). The findings of model 6, which examined the importance of exchange in learning groups during the digital semester, imply that the better developed the exchange in learning groups was during the digital semester, the higher students’ learning success (support for H 2.2). Finally, model 7 gives evidence that the higher students’ self-attributed digital competencies were, the higher students’ learning success (support for H 2.3). Compared to each other, with 6% explained variance per factor and regression coefficients of 0.25, the explanatory power was relatively high for the living situation and the exchange in learning groups. Surprisingly, self-attributed digital competencies seem to be distinctly less important for students’ learning success. Even during the digital semester, this factor explained not more than 2% of the given variance in students’ satisfaction with their learning success. Model 8 confirms the findings of model 5 to model 7: Even when controlling for the other factors, the explanatory power of every single factor remained stable. In sum, with the student-related aspects, 10% of the given variance in students’ satisfaction with their learning success could be explained. In comparison, the teacher-related aspects, with 14% explained variance, seem to have a higher explanatory power (Table 2).

#### Overall model

In the last step of our regression analysis, we tested all predictors on both levels—on the teacher level as well as on the student level—simultaneously (M 9, Table 4).

With 18.6% explained variance, the explanatory power of the overall model can be rated as moderate (Table 4). All included predictors show a positive and significant effect on students’ learning success. As the separate analyses of the teacher-related aspects have already suggested, the share of videoconferences/webinars (digital teaching situation) seems to be less important for students’ learning success (H 1.1); in the overall model, the regression coefficient of this aspect remained relatively small. In our model, the most important predictor for students’ learning success, with a regression coefficient of 0.30, was teachers’ digital competencies. On the student level, the factors most highly related to students’ learning success were a suitable living situation and the exchange within study groups.

### Learning success in different digital settings

To illustrate the overall impact of different digital conditions on the teacher level as well as on the student level, we finally present a simulation of students’ learning success under varying scenarios. On the one hand, we differentiated students at the teacher level, specifically, those students who had worse digital teaching conditions (no course was offered as videoconferences/webinars; no course offered direct interaction opportunities; not satisfied with teachers’ digital competencies) and students with perfect digital teaching conditions (all courses were offered as videoconferences/webinars; all courses offered direct interaction opportunities; very satisfied with teachers’ digital competencies). On the other hand, we differentiated the degree of digitalization on the student level for two student groups: students with worse individual digital learning conditions (the living situation was not suitable for many forms of digital learning; exchange within study groups became more difficult during the Corona pandemic; no digital competencies at all) and students with perfect individual digital learning conditions (the living situation was suitable for many forms of digital learning; exchange within study groups became easier during the Corona pandemic; high digital competencies).

**Fig. 2:**
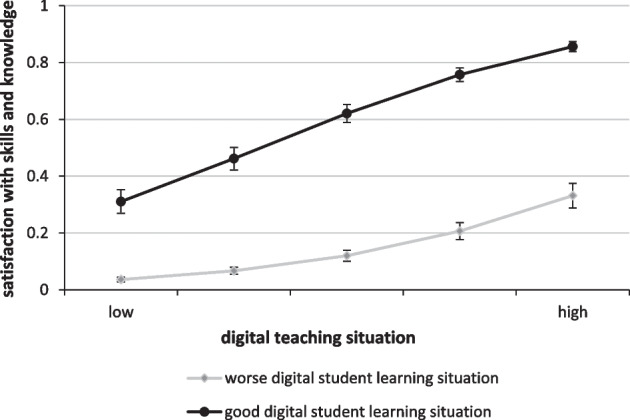
Simulation of students’ satisfaction with acquired knowledge and skills by different degrees of digitalization (predicted probabilities). Data: “Studying in Germany in Times of the Corona Pandemic”

Figure 2 shows that students' learning success increased with the degree of digitalization on the teacher level, and this occurred regardless of the conditions at the student level. Furthermore, the digital learning situation on the student level strongly affected students’ satisfaction with their learning success. Students with perfect learning conditions on the student level had a 28–53% higher probability of being satisfied with their acquired knowledge and competencies.

Students with perfect digital conditions on the teacher level as well as on the student level had the highest probability of being satisfied with their acquired knowledge and competencies (82%). In contrast, the probability of being satisfied with learning success tended to be zero when the digital conditions were not met, either on the student level or the teacher level.

## Discussion and conclusion

The present study addressed the question of how student- as well as teacher-related aspects contribute to the success of digital teaching at German universities during the pandemic in the summer of 2020. The analysis was based on the theoretical assumptions of the *theory of transactional distance* (Moore, 2018), which suggests that the success of digital teaching highly depends on the facilitation of dialogue, structure, and learner autonomy and that reducing the transactional distance shall induce higher learning success. In this study, we distinguished three variables each on the student- and the teacher-related level as indicators for the provision of interaction, the structural conditions allowing for digital teaching, and the digital competencies of teachers and students. By applying a set of multivariate regression, the results of the present study shed light on the role of the very specific conditions under which successful digital teaching can take place.

Overall, the core findings of this study can be summarized as follows: First, the scenario analysis revealed that both levels (e.g., student- as well teacher-related aspects) need to be considered in order to enable successful digital teaching and learning in higher education. In this sense, our findings provide initial guidance on which aspects institutions of higher education should focus on when developing or updating their digitalization strategies. Second, in accordance with collaborative learning approaches (for an overview, see Cleveland-Innes et al., 2018; Harasim, 2017; Jeong & Hmelo-Silver, 2016), a key factor for learning success appears to be enabling peer-to-peer interactions. This finding supports our prediction that the possibility of engaging in interactive learning activities is crucial for students’ learning experience, as it might reduce the perception of transactional distance and allow for social exchange. This coincides with previous findings emphasizing the role of social factors in distance learning environments (Nortvig et al., 2018). Finally, the strongest predictor of students’ learning success turned out to be the (perceived) digital competencies of the teachers. Studies in other European countries point out how important digital competence is for university teaching and how little it has been developed to date (Amhag et al., 2019; Sánchez-Cruzado et al., 2021). This finding clearly emphasizes that teachers must be qualified to address the very specific challenges of teaching in digital contexts and indicates that universities may need to implement more teacher qualification programs.

The findings of this study are based on cross-sectional data analysis and, thereby, help us to better understand the educational situation at German universities during the very beginning of the pandemic in the summer 2020. However, this methodological approach does not allow for any causal interpretation of the direction of the observed effects. For instance, the effect of teachers’ digital competencies on students’ learning success could also be explained in the opposite way: Students who are successful learners might be more willing to evaluate their teachers positively. Consequently, future research should supplement the present work via additional analysis in a longitudinal study design with panel data.

With respect to the measurements of the study, we note that learning success was merely operationalized by students’ self-ratings. Although self-reports are most frequently captured as dependent variables and are important indicators of learning success and student satisfaction (Kümmel et al., 2020), additional test-based competence measurements would allow us to draw a more holistic picture and would help us differentiate between students’ perception of what they might have learned (which could be confounded with other variables, such as interest in the topic or prior knowledge) and their actual learning gain.

Furthermore, this study focused exclusively on a subset of variables as indicators for the three core elements of the transactional distance theory (Moore, 1997, 2018). However, although these indicators seem to be elementary for analyzing the conditions of successful digital learning and teaching at German higher education institutions, capturing the whole theory is more complex. Consequently, future research would benefit from addressing this complexity by including further measures to represent the theoretical considerations more systematically and to enable the empirical testing of the entire theoretical model. In future studies, it seems crucial to further analyse the relationship between these independent variables. For example, by addressing the role of synchronous teaching for active learning opportunities in courses and interactive exchange within study groups.

Regarding *structure* and *learner autonomy,* in particular*,* a more in-depth analysis would be interesting. For example, future studies could investigate whether teacher responsiveness to a student’s needs and preferences affects learning success or how certain features of online teaching should be designed to promote independent learning.

Beyond this, the present study was based on measures of digital competencies reported by students (who reported on their digital competencies and those of their teachers), but the amount of detail collected in the survey was very broad. Although the survey information allowed us to make initial and crucial insights into the role that general digital competencies might play in successful teaching conditions, to derive specific courses of action that can be applied to university students and teachers, more fine-grained information on the multiple facets of competencies is needed. For instance, following the European Competence Framework, digital competence comprises at least five different subskills, ranging from presentation and communication skills to reflection and problem-solving skills (Carretero et al., 2017). In this vein, specific frameworks for educators also include media didactical skills (Caena & Redecker, 2019), which can be assumed to influence ratings of university teachers’ digital competencies. Beyond this, applying more detailed measures/instruments could also contribute to identifying other variables that might be confounded with these competencies in the context of digital teaching (other variables that might include, for instance, attitudes toward digital teaching, technology acceptance, and/or more general didactical competencies). Additionally, the present study did not consider the potential impact that certain subject areas might be associated with more/less digital competencies of both teachers and students. In this regard, it would be interesting to investigate whether certain didactic concepts of online learning can successfully reduce the transactional distance in specific domains, thereby helping us better understand which specific didactic concepts are required for a particular subject area. This needs to be addressed in future studies.

Finally, one must consider that our analysis was performed on the entire group of surveyed students and did not look at subgroups. For example, it could be that international students find recorded teaching units especially helpful because they can rewatch the lessons later on with the possibility to pause and translate if they do not understand something. As another example, students with children or care responsibilities may find asynchronous courses more compatible with their schedules.

Taken together, one might wonder how the findings of this study can contribute to a better understanding of the conditions of digital teaching in the future. Overall, with more than 18,000 student participants—even after the removal of around 10,000 subjects due to missing data, which could be related to the length of the survey and the often difficult personal circumstances of students in the context of the pandemic—our analysis was based on a large and representative dataset. Thus, we were able to identify and test central factors and determine their role in digital teaching and learning contexts. For instance, the findings highlight that successful learning strongly relies on a social component; importantly, providing space for interaction and exchange can serve purposes on different levels (e.g., cognitive and emotional; Mayweg-Paus et al., 2021). While these peer-to-peer interactions may influence the socio-emotional level differently when students’ circumstances change again (e.g., when students are able to meet face-to-face), the effects at the cognitive level should remain. However, as shown in the meta-analysis by Means et al. (2013), simply facilitating synchronous communication in online settings (such as in break-out rooms) does not positively impact students’ learning; rather, teachers need to provide further instructional guidance and support to assist students as they engage in deep online collaborative learning processes and acquire knowledge (Chen et al., 2018).

Also, we need to acknowledge that so-called emergency remote teaching (Hodges et al., 2020) is and should not be compared to online teaching, which is based on well-grounded didactical considerations. Apparently, designing teaching and learning scenarios in the digital sphere must go beyond the mere transfer of face-to-face didactical principles and should take into account the very specific conditions of online environments. In this regard, we can draw on an older research tradition that offers empirically tested scientific knowledge and, therefore, should enable evidence-based procedures and decisions in the context of online teaching and learning (Bernard et al., 2014; Means et al., 2013).

Furthermore, although the pressing circumstances of the pandemic have forced university teachers and students to immediately shift into the digital realm, at the same time it might increase openness and encourage universities to implement digital teaching and learning in the future. One important starting point would be for universities to systematically develop their lecturers’ and students’ digital competencies and to further invest in such programs. Thus, aside from all of the pandemic’s negative effects, it may help motivate higher education systems to create a functional and thought-through digitalization process.

## Supplementary Information

**Additional** **file 1.** Online Appendix Robustness Check.

## Data Availability

Lörz, M., Zimmer, L. M., Marczuk, A., Becker, K., Ehrhardt, M., Hinz, T., Meyer, J., Multrus, F., Naumann, H., Schirmer, H., Strauß, S. & Willige, J. (2020). Studieren in Zeiten der Corona-Pandemie. Datenerhebung: 2020. Version: 1.0.0. Datenpaketzugangsweg: Remote-Desktop-SUF. Hannover: FDZ-DZHW. Datenkuratierung: Daniel, A. https://doi.org/10.21249/DZHW:sitco2020:1.0.0.

## Footnotes

1 More detailed information on the technical procedure can be found in the technical report of the SITCO data set (https://doi.org/10.21249/DZHW:sitco2020:1.0.0).
