# Supplementary material for: Digital studying in times of COVID-19: teacher- and student-related aspects of learning success in german higher education
Source: Int J Educ Technol High Educ. 2023 Mar 3;20(1):12. doi: 10.1186/s41239-023-00382-w (PMC9981250; doi:10.1186/s41239-023-00382-w)
Supplement: Supplementary file 1 — Additional file 1. Online Appendix Robustness Check. [file 41239_2023_382_MOESM1_ESM.pdf]

Online Appendix

Table A-1

Means, standard deviations, minimum, maximum, and correlations, N= 18,262

	Variable	% (SD)	Min	Mx	Phi
**Dependent variable**	(Very) satisfied with the **knowledge and competencies acquired to date** (=1)	39% (.49)	0	1	-
**Teacher-related predictors**	**A1:** More than half of the courses took place as a **videoconference/webinar** (=1)	52% (.50)	0	1	.08***
	**A2:** More than half of the courses offered active **interaction opportunities** (=1)	55% (.50)	0	1	.15***
	**A3:** (Very) satisfied with **teacher's digital competencies** (=1)	42% (.50)	0	1	.26***
**Student-related predictors**	**B1: Living situation** is (very) suitable for many forms of digital learning (=1)	68% (.47)	0	1	.15***
	**B2: Exchange within study groups** during the Corona pandemic became (much) easier (=1)	6% (.23)	0	1	.08***
	**B3:** Possessing **digital competencies** (to a large extent) (=1)	69% (.46)	0	1	.10***

* $p < 0.05$, ** $p < .01$, *** $p < .001$. *Data*: "Studying in Germany in Times of the Corona Pandemic"

Table A-2

Effect of the teacher-related aspects on students' satisfaction with acquired knowledge and competencies

	M 1	M 2	M 3	M 4
More than half of the courses took place as a videoconference/webinar	.061***			.014
More than half of the courses offered active interaction opportunities		.128***		.082***
(Very) satisfied with teacher's digital competencies			.240***	.223***
N	18,262	18,262	18,262	18,262
Pseudo R^2	0.016	0.026	0.057	0.063
Log-likelihood	-12242.76	-12119.15	-11725.65	-11649.96

* $p < 0.05$, ** $p < .01$, *** $p < .001$, Binary Logistic Regression, AME reported; models controlled for gender, subject group, university semester, and pursued degree. Data: "Studying in Germany in Times of the Corona Pandemic".

Table A-3

Effect of the student-related aspects on students' satisfaction with acquired knowledge and competencies

	M 5	M 6	M 7	M 8
Living situation is (very) suitable for many forms of digital learning	.164***			.156***
Exchange within study groups during the Corona pandemic became (much) easier		.170***		.147***
Possessing digital competencies (to a large extent)			.093***	.081***
N	18,262	18,262	18,262	18,262
Pseudo R²	0.031	0.017	0.018	0.039
Log-likelihood	-12043.73	-12223.56	-12209.92	-11948.97

* p < 0.05, ** p < .01, *** p < .001, Binary Logistic Regression, AME reported; models controlled for gender, subject group, university semester, and pursued degree. Data: "Studying in Germany in Times of the Corona Pandemic".

Table A-4

Effect of teacher-related and student-related aspects on students' satisfaction with acquired knowledge and competencies

		M 9
**Teacher level**	More than half of the courses took place as a videoconference/webinar	.017
	More than half of the courses offered active interaction opportunities	.072***
	(Very) satisfied with teacher's digital competencies	.200***
**Student level**	Living situation is (very) suitable for many forms of digital learning	.119***
	Exchange within study groups during the Corona pandemic became (much) easier	.114***
	Possessing digital competencies (to a large extent)	.070***
N		18,262
Pseudo R²		0.079
Log-likelihood		-11443.78

* p < 0.05, ** p < .01, *** p < .001, Binary Logistic Regression, AME reported; models controlled for gender, subject group, university semester, and pursued degree. Data: "Studying in Germany in Times of the Corona Pandemic".
